# Effects of an Animated Blood Clot Technology (Visual Clot) on the Decision-Making of Users Inexperienced in Viscoelastic Testing: Multicenter Trial

**DOI:** 10.2196/27124

**Published:** 2021-05-03

**Authors:** Sadiq Said, Tadzio Raoul Roche, Julia Braun, Micheal Thomas Ganter, Patrick Meybohm, Johannes Herrmann, Kai Zacharowski, Florian Jürgen Raimann, Florian Piekarski, Eva Rivas, Manuel López-Baamonde, Donat R Spahn, Christoph Beat Nöthiger, David Werner Tscholl

**Affiliations:** 1 Institute of Anesthesiology University Hospital Zurich University of Zurich Zurich Switzerland; 2 Department of Epidemiology Epidemiology, Biostatistics and Prevention Institute University of Zurich Zurich Switzerland; 3 Institute of Anesthesiology and Pain Therapy Cantonal Hospital Winterthur Winterthur Switzerland; 4 Department of Anesthesiology, Intensive Care, Emergency, and Pain Medicine University Hospital Wuerzburg University of Wuerzburg Wuerzburg Germany; 5 Department of Anesthesiology, Intensive Care Medicine, and Pain Therapy University Hospital Frankfurt Goethe University Frankfurt Frankfurt Germany; 6 Department of Anesthesiology, Intensive Care Medicine, and Pain Therapy Hospital Clinic of Barcelona University of Barcelona Barcelona Spain

**Keywords:** avatar technology, coagulation management, hemostasis, intuitive design, rotational thromboelastometry, user-centered design, Visual Clot, testing

## Abstract

**Background:**

Viscoelastic test–guided coagulation management has become increasingly important in assessing hemostasis. We developed Visual Clot, an animated, 3D blood clot that illustrates raw rotational thromboelastometry (ROTEM) parameters in a user-centered and situation awareness–oriented method.

**Objective:**

This study aimed to evaluate the applicability of Visual Clot by examining its effects on users that are novices in viscoelastic-guided resuscitation.

**Methods:**

We conducted an investigator-initiated, international, multicenter study between September 16, 2020, and October 6, 2020, in 5 tertiary care hospitals in central Europe. We randomly recruited medical students and inexperienced resident physicians without significant prior exposure to viscoelastic testing. The 7 participants per center managed 9 different ROTEM outputs twice, once as standard ROTEM tracings and once as the corresponding Visual Clot. We randomly presented the 18 viscoelastic cases and asked the participants for their therapeutic decisions. We assessed the performance, diagnostic confidence, and perceived workload in managing the tasks using mixed statistical models and adjusted for possible confounding factors.

**Results:**

Analyzing a total of 630 results, we found that the participants solved more cases correctly (odds ratio [OR] 33.66, 95% CI 21.13-53.64; *P*<.001), exhibited more diagnostic confidence (OR 206.2, 95% CI 93.5-454.75; *P*<.001), and perceived less workload (coefficient –41.63; 95% CI –43.91 to –39.36; *P*<.001) using Visual Clot compared to using standard ROTEM tracings.

**Conclusions:**

This study emphasizes the practical benefit of presenting viscoelastic test results in a user-centered way. Visual Clot may allow inexperienced users to be involved in the decision-making process to treat bleeding-associated coagulopathy. The increased diagnostic confidence, diagnostic certainty, reduced workload, and positive user feedback associated with this visualization may promote the further adoption of viscoelastic methods in diverse health care settings.

## Introduction

Since Hartert invented viscoelastic testing in 1948 [1] and its later clinical introduction in the 1980s [2,3], viscoelastic coagulation monitoring has become increasingly important in assessing acute bleeding in patients. To this end, several leading guidelines have proposed the use of viscoelastic-guided transfusion algorithms [4,5]. Compared to standard laboratory coagulation assays, rotational thromboelastometry (ROTEM) is faster [6,7], reduces inappropriate blood transfusions [8], and is more cost-efficient overall [9]. Further, previous studies showed that goal-directed viscoelastic hemostatic resuscitation improved patient outcomes in various different surgical specialties [9-13]. However, despite its evident importance, widespread acceptance, and increasing use, correctly interpreting ROTEM outputs remains a significant challenge for inexperienced physicians.

**Figure 1:**
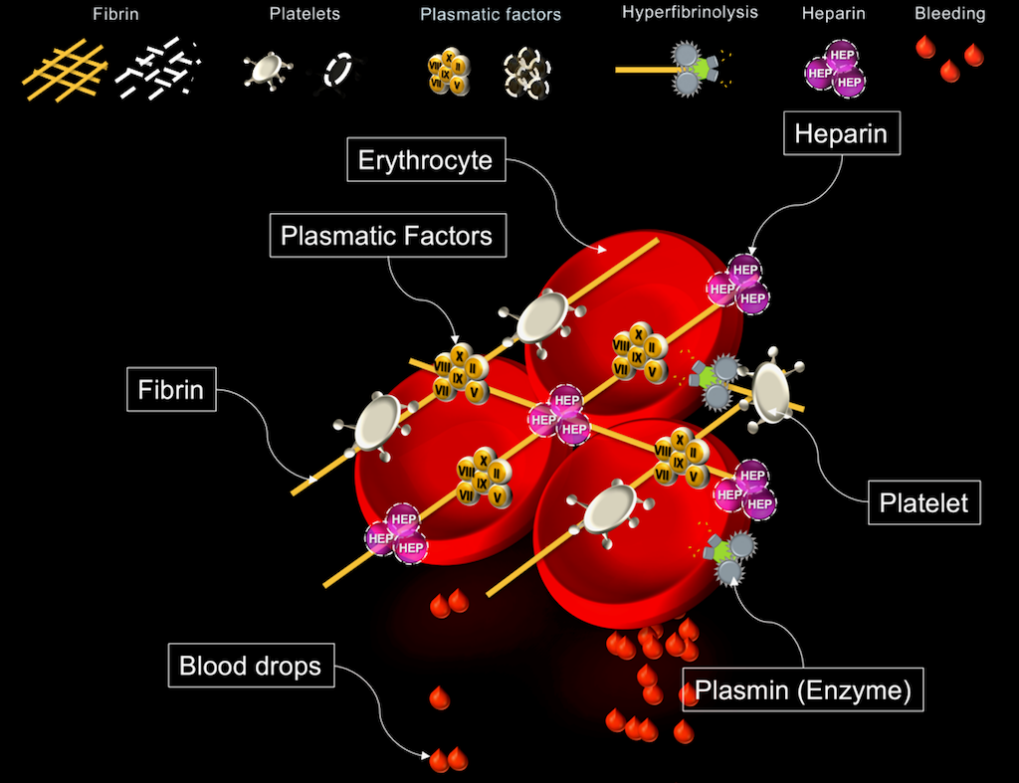
Example of the animated blood clot, Visual Clot. Blood drops are shown in cases of deficient hemostatic components.

Hence, our research department aimed to simplify viscoelastic test outputs by developing Visual Clot technology. This animated, 3D blood clot illustrates the raw ROTEM parameters in a user-centered and situation awareness–oriented method. Visual Clot displays different coagulation components as either present or absent based on empirical ROTEM cutoff values, without making the final decision for the user. Figure 1 shows the functionality of the Visual Clot technology. In a previous, prospective, dual-center study, Visual Clot helped anesthesia and intensive care physicians in Germany and Switzerland to improve their therapeutic decisions in coagulation management [14]. In a computer-based environment, the physicians were faster, exhibited more confidence, and experienced less workload in managing the hypothetical ROTEM outputs [14]. After their initial experiences, the same physicians considered Visual Clot as intuitive, easy to learn, and useful for the decision-making process [15].

In contrast to the previous research [14,15], this study aimed to evaluate the applicability of Visual Clot by examining its effects on users that are novices in viscoelastic-guided resuscitation. Without giving any instructions on analyzing or interpreting viscoelastic results, we tested the performance of using Visual Clot compared to that of using standard ROTEM readings. We hypothesized that these inexperienced users would solve more simulated bleeding scenarios correctly, with more diagnostic confidence and less perceived workload using this avatar technology. The results of this study may support the concept of Visual Clot technology and demonstrate its potential for involving inexperienced physicians in coagulation diagnostics and management. Moreover, this study promotes the further development of user-centered, situation awareness–oriented visualization technologies.

## Methods

This was an investigator-initiated, computer-based, within-subject, international multicenter study comparing standard ROTEM results with a corresponding animated viscoelastic visualization in simulated bleeding situations. We conducted this study between September 2020 and October 2020 in 5, large, tertiary care hospitals. The Cantonal Hospital Winterthur and University Hospital Zurich in Switzerland, the University Hospital Frankfurt and University Hospital Wuerzburg in Germany, and Hospital Clinic de Barcelona in Spain participated as different centers. The leading ethics committee in Zurich waived this study as it was not within the scope of the human research act (no. BASEC-Nr. Req-2020-00906). The other centers in Germany and Spain also waived ethical approval. All participants agreed in writing to the further use of obtained data for research purposes.

### Study Procedure

For participants, we included medical students in their last or penultimate year of study and inexperienced resident physicians without significant prior exposure to viscoelastic coagulation testing. Further inclusion criteria were that they had never seen Visual Clot before and had no or minimal self-declared ROTEM skills. All participants worked in the respective hospitals. Participant selection was according to availability in clinical practice and inexperience in viscoelastic resuscitation. This subject population was completely different from that of the previous Visual Clot study [14], in which we investigated the technology’s effect on experienced anesthesia and intensive care physicians.

**Figure 2:**
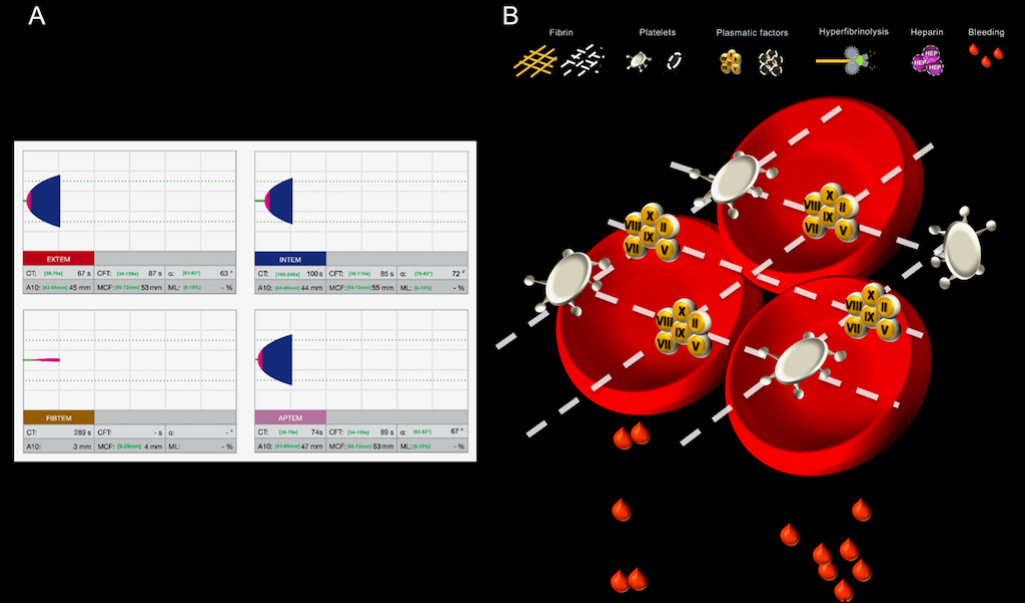
Example of a standard rotational thromboelastometry (ROTEM) presentation with a corresponding Visual Clot. Adhering to the coagulation algorithm used at the University Hospital Zurich, this hemostatic state represents fibrin deficiency.

We prepared 9 different bleeding scenarios that indicated specific coagulation disorders or a normal hemostatic state. In Multimedia Appendix 1, we provide a detailed list of all scenarios including their recommended therapeutic options. We showed each scenario twice, either as standard ROTEM readings or as the corresponding Visual Clot. Figure 2 shows an example of a standard ROTEM presentation with a corresponding Visual Clot, while Multimedia Appendix 2 shows the Visual Clot instructional video. We programmed Visual Clot according to the Zurich coagulation algorithm (Multimedia Appendix 3), which was validated in clinical practice [11]. When showing the bleeding scenarios as standard ROTEM readings, we provided the participants with the same coagulation algorithm’s normal values [11]. At the beginning of the study, we first asked the participants to fill out a demographic survey on personal data, such as age, gender, and educational level. We then randomly presented the 9 different coagulation scenarios twice without any instructions on how to interpret them. We used online software [16] to randomize the sequence of these 18 scenarios, providing each participant with a unique set. We showed all ROTEM readings or the respective Visual Clot on an Apple MacBook (Apple Inc). Using exclusively the respective viscoelastic results presentation, we asked the participants to choose their targeted therapeutic recommendations from a total of 6 given answer options. It was possible that multiple therapeutic interventions were necessary for sufficient treatment. We provided the answers as checkboxes in multiple choice form using the app iSurvey (Harvest Your Data) displayed on an iPad (Apple Inc) [17]. We encouraged all participants to submit their therapeutic recommendations as quickly and accurately as possible. After each scenario, the participants rated their diagnostic confidence and perceived workload in fulfilling the given task. At the end of the study session, we asked the participants to rate 4 statements on a 5-point Likert scale (from strongly disagree to strongly agree). These statements aimed to obtain a deeper understanding of the participants’ opinions about Visual Clot technology.

### Outcomes

The primary outcome of this study was performance, a binary outcome defined as correctly or incorrectly solved scenarios. In scenarios with multiple required therapeutic elements, we considered them to be correctly managed if the participant selected all the correct therapeutic options and no incorrect ones. As secondary outcomes of this study, we assessed the diagnostic confidence binary as unconfident or confident, and the perceived workload using the raw NASA (National Aeronautics and Space Administration) Task Load Index questionnaire. This subjective workload assessment tool has been validated in many different areas, including health care [18-23]. The raw questionnaire defines the total perceived workload as the arithmetic mean of 6 different, workload-associated subscores [23-25]. This study did not investigate the physical demand subscore, as our tasks were not physically challenging.

### Statistical Analysis

As a first unadjusted analysis, we applied the McNemar test to compare the numbers of correctly and incorrectly solved cases with ROTEM and Visual Clot. We calculated mixed logistic regression models with a random intercept for each participant for the binary outcome variables, performance as correct or incorrect, and confidence as unconfident or confident. Further, we calculated a linear mixed model with a random intercept per participant for the continuous outcome regarding perceived workload as measured by the overall NASA Task Load Index scores. Apart from the variable denoting the respective viscoelastic modality (ROTEM vs Visual Clot), we adjusted all models for the center, gender, and job experience as confounders. We did not include the respective scenario as a confounding factor because the order was completely randomized for each participant and would not have consequently influenced the overall results.

As we expected similar or greater performance differences as in the previously published Visual Clot study with experienced ROTEM users, we conducted an a priori sample size calculation based on these previous results [14]. We had reported a median of 44% (317/720) correct decisions for ROTEM and 100% (720/720) correct decisions using the Visual Clot. Based on the McNemar test and assuming the proportions of correct solutions were found in the pilot study, we calculated a sample size of 15 participants to achieve a significance level of 5% and a power of 90%. As the data collection was very cost- and time efficient, we decided to include 7 participants in each center to adjust the analyses for the different centers.

We examined all data using R Version 3.6.2 (R Foundation for Statistical Computing) and created graphs using GraphPad Prism Version 9.0.0 (GraphPad Software Inc). Statistical significance was considered at a *P* value <.05.

## Results

Between September 16, 2020, and October 6, 2020, each of the 5 study centers included 7 participants, amounting to 35 participants in total. We exposed each participant to 18 coagulation management scenarios, providing ROTEM results in 9 cases and Visual Clot in the remaining corresponding cases. No data were excluded in the final analysis. We investigated a total of 630 results, 315 per viscoelastic output modality. Regarding the participants, 49% (17 of 35) were female and none had previous contact with Visual Clot technology. Table 1 displays further study and participant characteristics. In Multimedia Appendix 4, we provide the full statistical analysis of this study.

**Table 1:** Study and participant characteristics (N=35).

Characteristic			Value
Study centers, n			5
Age (years), median (IQR, range)			28 (25-32, 24-36)
Self-rated theoretical ROTEM^a^ knowledge^b^, median (IQR, range)			0 (0-10, 0-20)
Number of ROTEMs interpreted per year^c^, median (IQR, range)			0 (0-0, 0-6])
**Experience, n (%)**			
	Penultimate year of medical studies	3 (9%)	
	Last year of medical studies	10 (29%)	
	First year resident physician	19 (54%)	
	Second year resident physician	2 (6%)	
	Third year resident physician	1 (3%)	

^a^ROTEM: rotational thromboelastometry.

^b^The self-rated ROTEM knowledge scale ranges from 0 (very low) to 100 (very high).

^c^The number of ROTEM interpreted per year ranges from 0 (very low) to 100 (very high).

**Figure 3:**
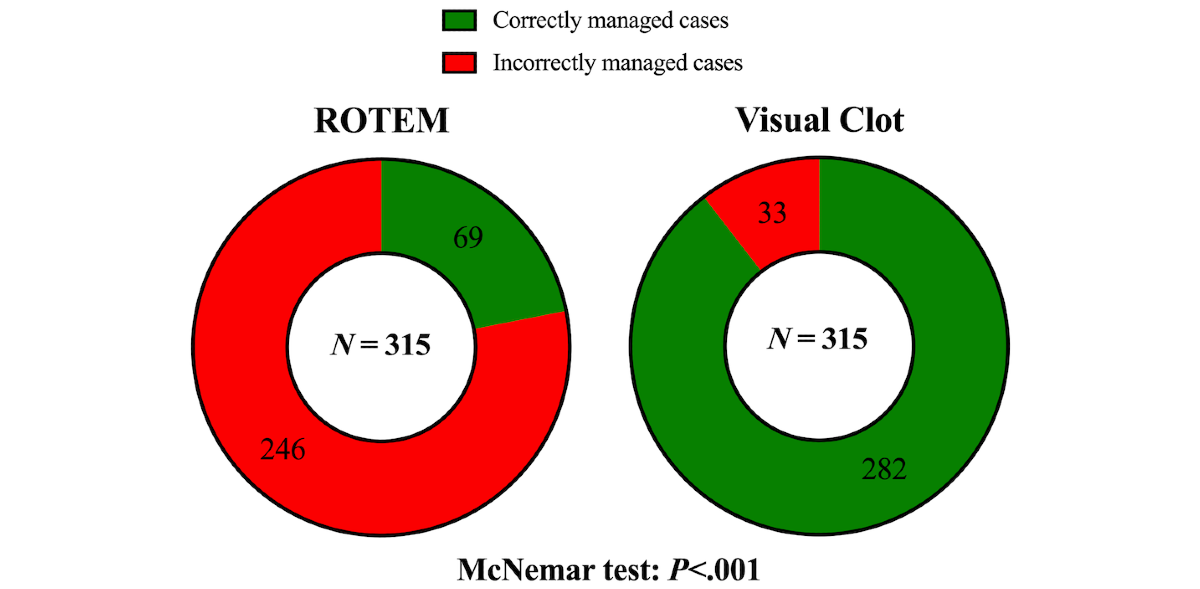
Donut charts displaying the binary performance defined as the number of correctly and incorrectly solved cases using standard ROTEM results (left donut) or Visual Clot (right donut). The unadjusted analysis using the McNemar test showed very strong evidence for a difference between the 2 viscoelastic modalities (*P*<.001). N=315 per viscoelastic modality. ROTEM: rotational thromboelastometry.

Regarding our primary outcome, binary performance, the mixed logistic regression provided very strong evidence for a difference between the 2 viscoelastic modalities. The odds of correctly solving the case were about 33 times as high when using Visual Clot compared to when using conventional ROTEM tracings (odds ratio [OR] 33.66, 95% CI 21.13-53.64; *P*<.001). In Figure 3, we illustrate the unadjusted comparison between the viscoelastic modalities using the McNemar test. There was no significant difference in performance between the genders (OR 0.63, 95% CI 0.38-1.03; *P*=.06) or the different study centers (all *P* values >.05; Cantonal Hospital Winterthur *P*=.41; University Hospital Frankfurt *P*=.65; University Hospital Würzburg *P*=.60; Hospital Clinic de Barcelona *P*=.69). The job experience, represented by the educational level, did not differ significantly in terms of performance either (all *P*>.05; last year of studies *P*=.59; first year of residency *P*=.64; second year of residency *P*=.96; third year of residency *P*=.75).

For the analysis of the participants’ diagnostic confidence ratings, our results showed very strong evidence in favor of Visual Clot. The odds of being diagnostically confident were about 200 times higher than when using ROTEM printouts (OR 206.2, 95% CI 93.5-454.75; *P*<.001). Regarding this outcome, no significant differences were not found for the ratings of genders (*P*=.96), centers (Cantonal Hospital Winterthur *P*=.10; University Hospital Frankfurt *P*=.42; University Hospital Würzburg *P*=.12; Hospital Clinic de Barcelona *P*=.08), or job experiences (last year of studies *P*=.73; first year of residency *P*=.97; second year of residency *P*=.32; third year of residency *P*=.18).

**Figure 4:**
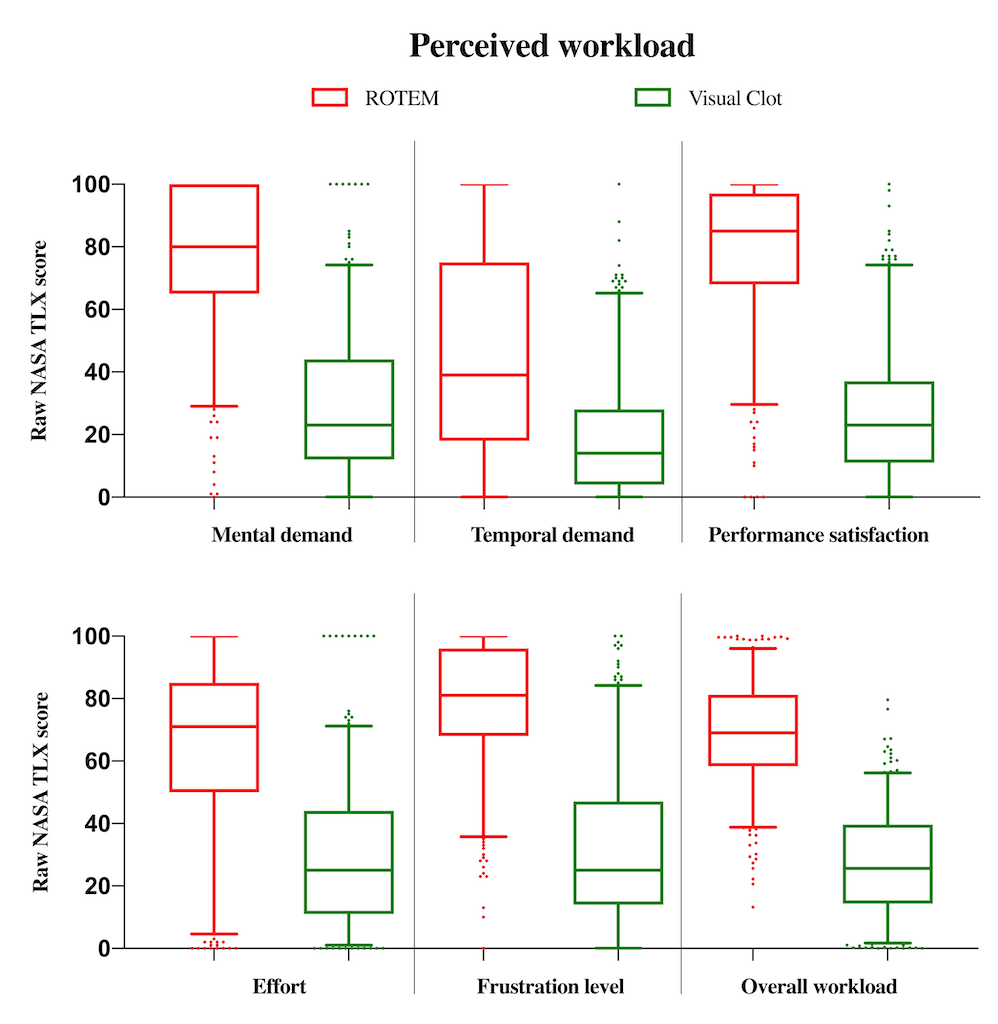
Boxplots representing the analysis of the participants’ perceived workload after using the respective viscoelastic modality. The overall workload and its subscores were evaluated using the modified, raw NASA Task Load Index questionnaire. Low workload scores correspond to low perceived workload. The box represents the first and third quartiles, with the line indicating the median. N=315 per viscoelastic modality. The whiskers represent the 5th and 95th percentile. NASA: National Aeronautics and Space Administration; ROTEM: rotational thromboelastometry; TLX: Task Load Index.

Finally, mixed linear regression yielded very strong evidence for a difference between the 2 viscoelastic modalities regarding perceived workload ratings. The overall raw NASA Task Load Index scores were on average about 40 points lower if Visual Clot was used, with a coefficient of –41.63 (95% CI –43.91 to –39.36l; *P*<.001). In Figure 4, we provide the analysis of the overall perceived workload and its subscores as boxplots.

**Figure 5:**
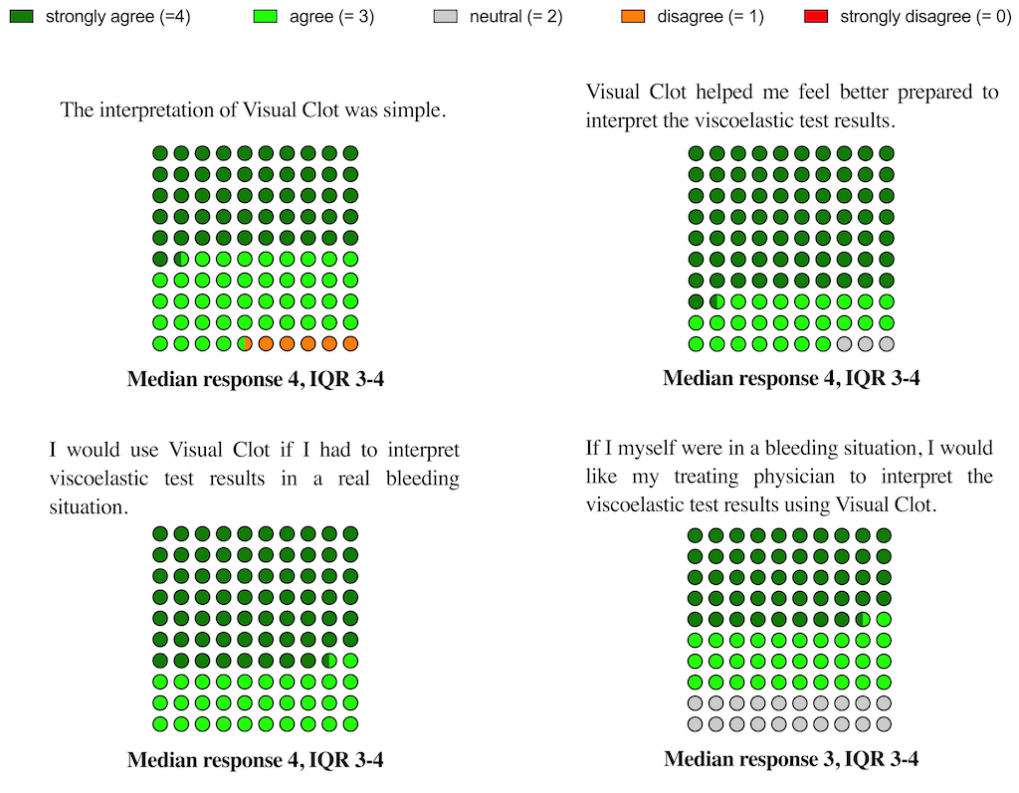
Graphical presentation of the participants’ rated survey statements as 10 x 10 parts of whole dot plots. Results are presented as median and IQR. N=35 in each rated statement.

Figure 5 illustrates the 4 statements regarding the participants’ opinions about Visual Clot and the results of their assessment. All following results are presented as agree (strongly agree or agree), neutral, or disagree (strongly disagree or disagree). Out of 35 participants, 34 (97%) agreed that the interpretation of Visual Clot was simple and 33 (94%) agreed that Visual Clot helped them feel better prepared to interpret viscoelastic test results. Moreover, all participants (35/34, 100%) agreed that they would use Visual Clot technology in a real bleeding situation, and 28 of 35 (80%) would want their treating physician to use Visual Clot if they were experiencing acute bleeding.

## Discussion

This study compared 315 within-subject therapeutic decisions of 35 participants who were inexperienced in viscoelastic-guided hemostatic resuscitation and who did not receive any instructions in interpreting the result printouts. Using the animated blood clot, Visual Clot, the participants interpreted more viscoelastic test results correctly than when using the standard ROTEM tracings. Moreover, they felt more confident in their therapeutic decision and perceived less workload using Visual Clot technology. None of the participants had any previous contact with the avatar-based presentation before attending the study. We examined the results using mixed models and adjusted for different confounders.

Analysis of the participants’ performance revealed that the participants selected the correct therapeutic options in over 90% (282 of 315) of the Visual Clot cases, while they made the same choices in only about 22% (69 of 315) using the corresponding ROTEM. This resulted in a relative risk ratio of about 4 and 33-times higher odds of correctly interpreting the test results when using Visual Clot technology. This positive effect persisted both across the different study centers and across the participants’ educational levels. These results are even more pronounced than those in the primary Visual Clot study, where we investigated viscoelastic-experienced physicians [14]. There, the odds of a correct therapeutic decision using the avatar technology were around 22 times increased. Visual Clot appears to have significant positive effects even without any instructions for interpretation and even for users with minimal viscoelastic-guided resuscitation knowledge.

Indeed, perceived usability is a prerequisite for user acceptance of innovative technologies [26]. Both users’ intuition (ie, unconscious reasoning) and the technology’s characteristics, such as shape, color, and presentation of its features, directly influence how people interact with unfamiliar devices [27]. Out of 35 participants, 33 agreed that interpreting Visual Clot was simple. In a previous study investigating physicians’ opinions on Visual Clot, the participants found the avatar useful, easy to learn, and intuitive [15]. Likewise, they mentioned that it allowed a faster overview of complex coagulation situations [15]. Visual Clot dichotomizes a disease state or the presence of substrates necessary for optimal clotting based on pathological, self-determinable ROTEM thresholds of an integrated, adequate coagulation algorithm. Indicating essential factors as present or absent and demonstrating them in a playful, user-centered manner reduces information complexity, leading to increased cognitive reception [28]. This binary illustration facilitates decision-making, may help to enforce local coagulation guidelines, and can reduce uncertainty in ambiguous situations through its clear presentation. On the other hand, the standard ROTEM printouts require that the physician understands the numerical results and the data-driven tracings in the context of the coagulation algorithm’s normal values to form a mental model of the current bleeding situation. This seems to cause more insecurity and incorrect therapeutic decisions that may affect the treatment of patients.

Our analysis showed a significant reduction of participants’ perceived workload scores when using Visual Clot than when using traditional ROTEM results. Furthermore, our analyses showed 200 times higher odds of being confident when using Visual Clot. Again, these results are more pronounced in these novice users than in the experienced anesthesiologists and intensive care specialists of the previous Visual Clot study [14]. In large hospitals, inexperienced resident physicians may be confronted with acute bleeding situations and coagulopathy even before they possess sufficient medical training in this field. This may cause a high-pressure working environment, which is known to degrade performance [29] and lead to fatigue from perceived work overload [30]. Staff well-being directly influences the prevalence of medical errors [31], and confidence positively affects performance [32]. We should strive to minimize workload and promote the staff’s diagnostic confidence to ensure better patient outcomes.

In this study, all of the participants agreed that they would use Visual Clot in a real bleeding situation. Further, 80% (28 of 35) agreed that they would want their treating physician to use this technology if they were experiencing acute bleeding themselves. It seems that the participants trust the technology and accept its application. However, we designed Visual Clot to complement the quantitative ROTEM data as a graphical representation, rather than to replace them.

This study had several limitations. Using a computer-based simulation design, we generated ROTEM printouts and corresponding Visual Clot animations that were clearly attributable to a coagulation disorder or normal hemostatic state. Viscoelastic results in real clinical bleeding may be less distinctive. Future studies are needed to confirm the results of this study in real bleeding-associated coagulopathic situations. However, simulation studies are considered an optimal environment to train and assess new methods [33]. Further, we performed this study in tertiary care hospitals in central Europe, and the results may differ elsewhere in the world. However, we consider this unlikely as all participants were novices to viscoelastic-guided management and therefore did not yet benefit from those large facilities’ medical training.

This study also possesses several strengths. The analyses were adequately powered due to the a priori sample size calculation. Furthermore, the within-subject comparisons may largely rule out alternative explanations for our findings. The multicenter design and balanced participant selection across the 5 study centers minimized selection bias.

This study emphasizes the relevance of designing viscoelastic test results in a user-centered, situation awareness–oriented method. The avatar-based blood clot presentation enabled users with no or minimal knowledge in viscoelastic-guided coagulation management and without any prior training to solve almost all coagulation scenarios correctly. It further improved the participants’ diagnostic confidence and reduced their perceived workload. Straightforward and confident interpretation may benefit new and experienced users in a wide range of treatment settings and promote the adoption of viscoelastic methods. The most significant benefits will likely be gained by inexperienced users and users who need to make quick decisions in stressful situations, such as on the battlefield, in spaceflight, or in the emergency room. The potential benefits of this technology and the emerging use of viscoelastic testing with its evidence-based importance justifies further investigation of Visual Clot in real clinical bleeding, with the ultimate aim of improving patient outcomes.

## Appendix

Multimedia Appendix 1 All scenarios including their recommended therapeutic options.

Multimedia Appendix 2 Instructional video showing the functionality of Visual Clot.

Multimedia Appendix 3 Visual Clot algorithm according to the local rotational thromboelastometry thresholds at the University Hospital Zurich.

Multimedia Appendix 4 Full statistical analysis of this study.

## Abbreviations

NASA: National Aeronautics and Space Administration
OR: odds ratio
ROTEM: rotational thromboelastometry
