# Supplementary material for: Effects of an Animated Blood Clot Technology (Visual Clot) on the Decision-Making of Users Inexperienced in Viscoelastic Testing: Multicenter Trial
Source: J Med Internet Res. 2021 May 3;23(5):e27124. doi: 10.2196/27124 (PMC8129883; doi:10.2196/27124)

Effects of an animated blood clot on the decision-making of users inexperienced in viscoelastic testing - Visual Clot technology: a multicenter trial

Sadiq Said, Tadzio Raoul Roche, Julia Braun, Michael Thomas Ganter, Patrick Meybohm, Johannes Herrmann, Kai Zacharowski,

Eva Rivas, Manuel Lopez-Baamonde, Florian Jürgen Raimann, Florian Piekarski, Donat Rudolf Spahn, Christoph Beat Nöthiger,

David Werner Tscholl

Heparin effect

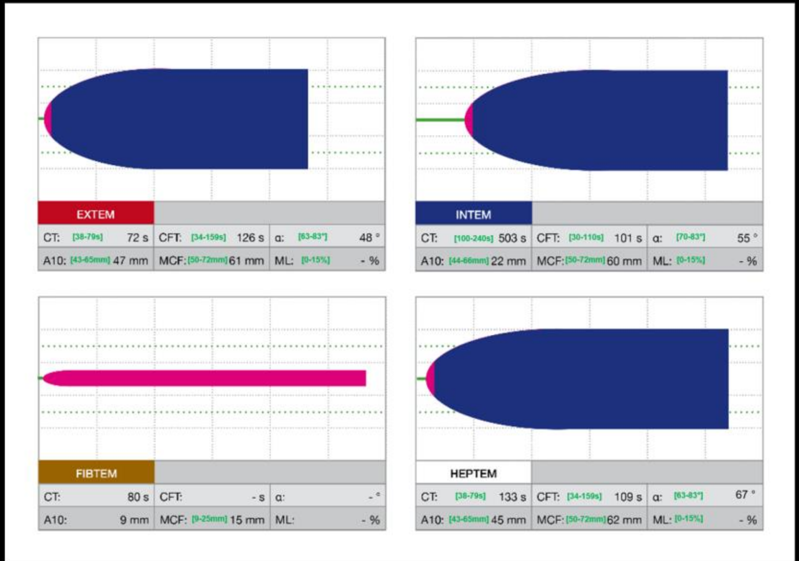

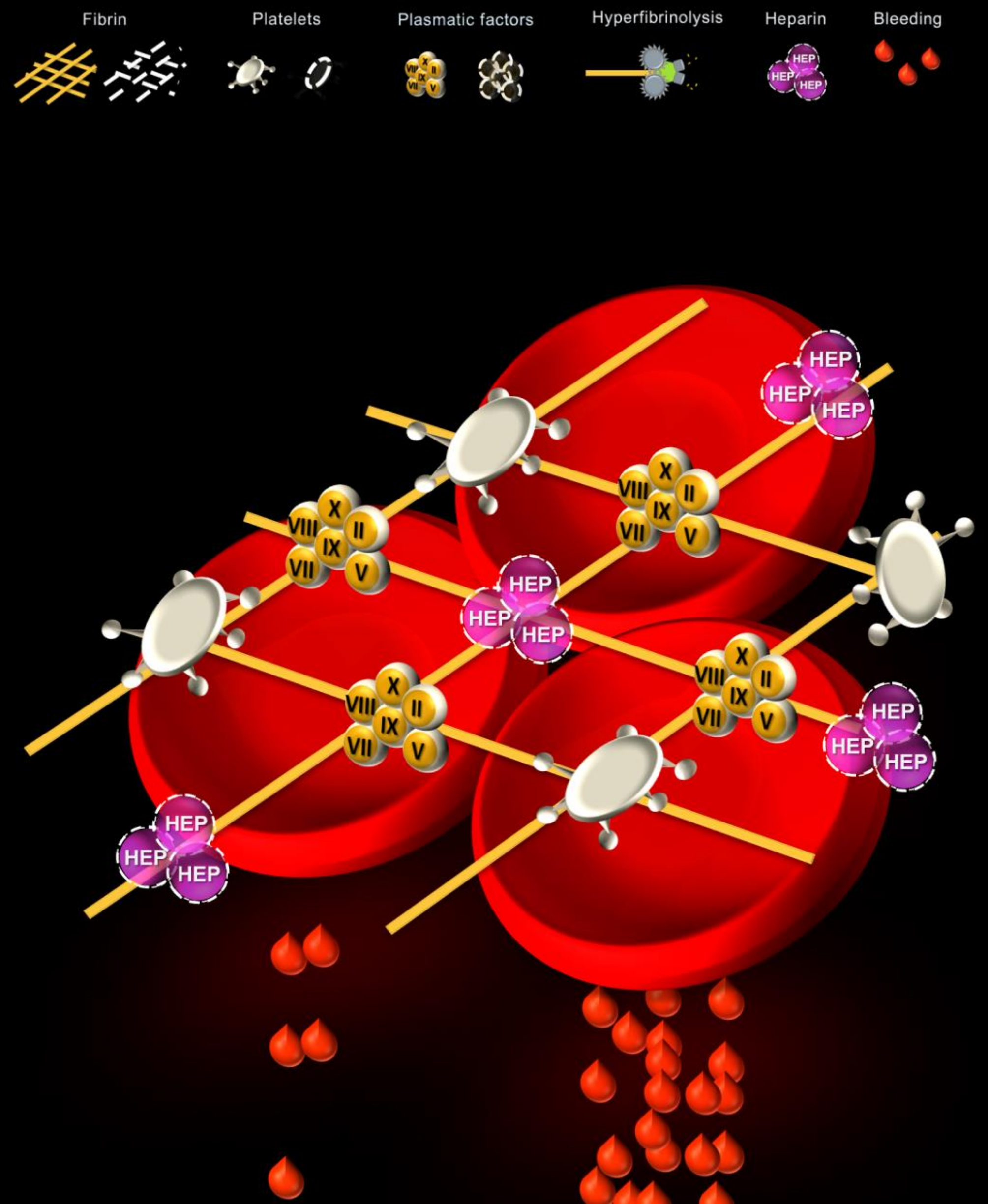

Solution:
Fibrinogen
Platelets
Anti-hyperfibrinolytic
Protamine
Plasmatic factors
No treatment required

Normal

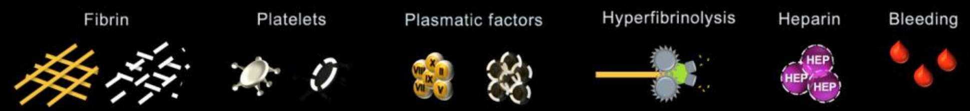

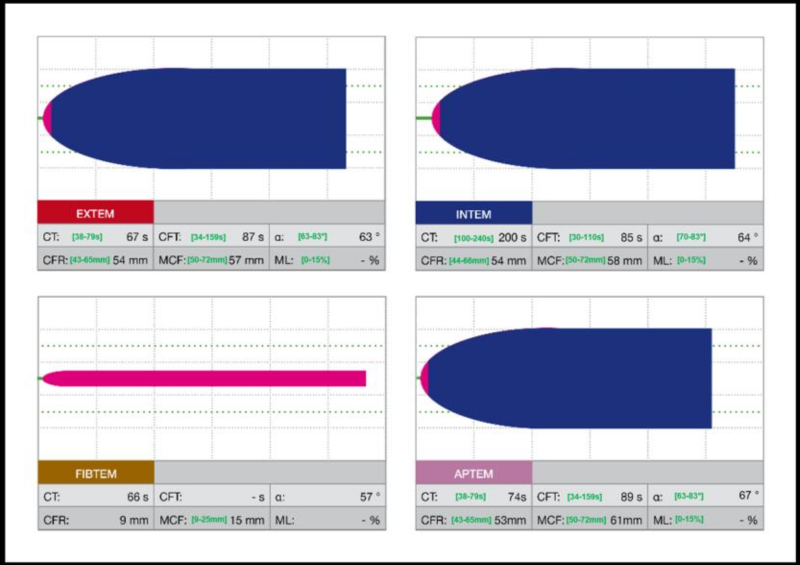

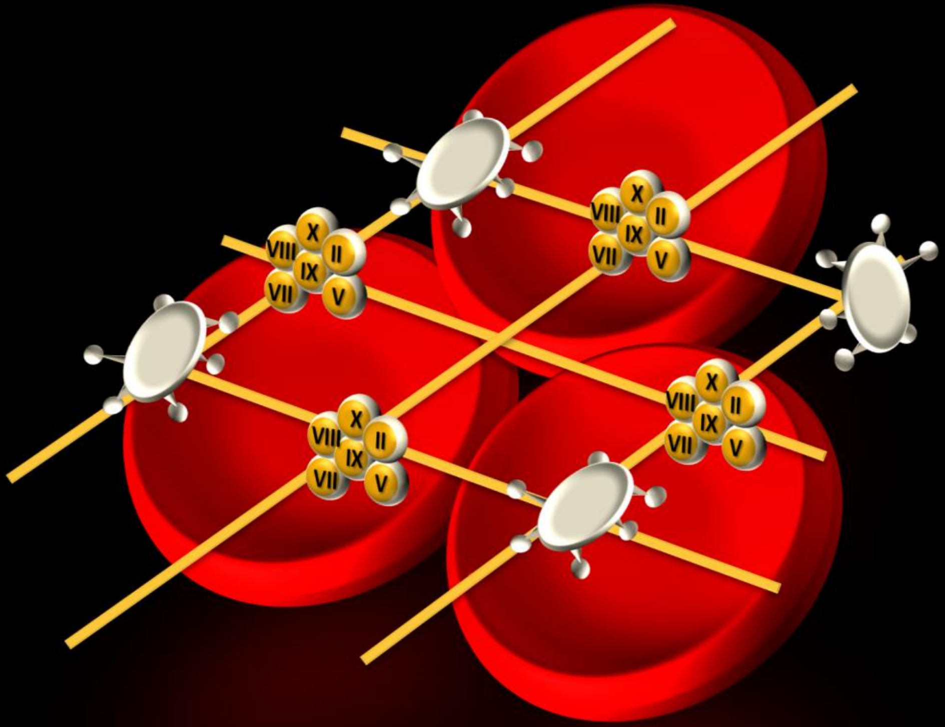

Solution:
Fibrinogen
Platelets
Anti-hyperfibrinolytic
Protamine
Plasmatic factors
No treatment required

Combined plasmatic factors, fibrin and platelets deficiency

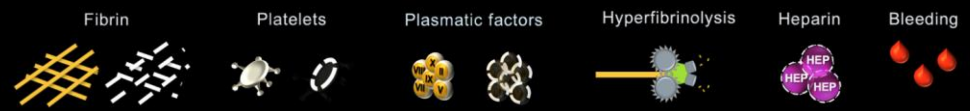

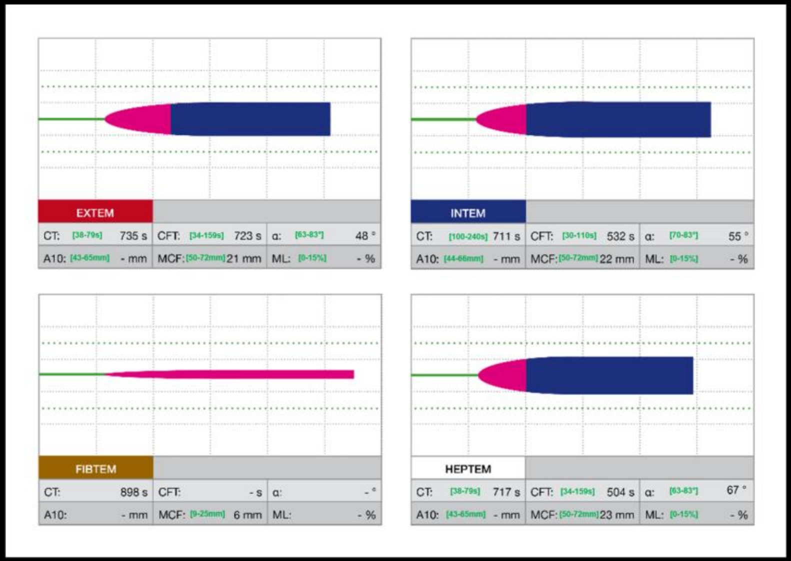

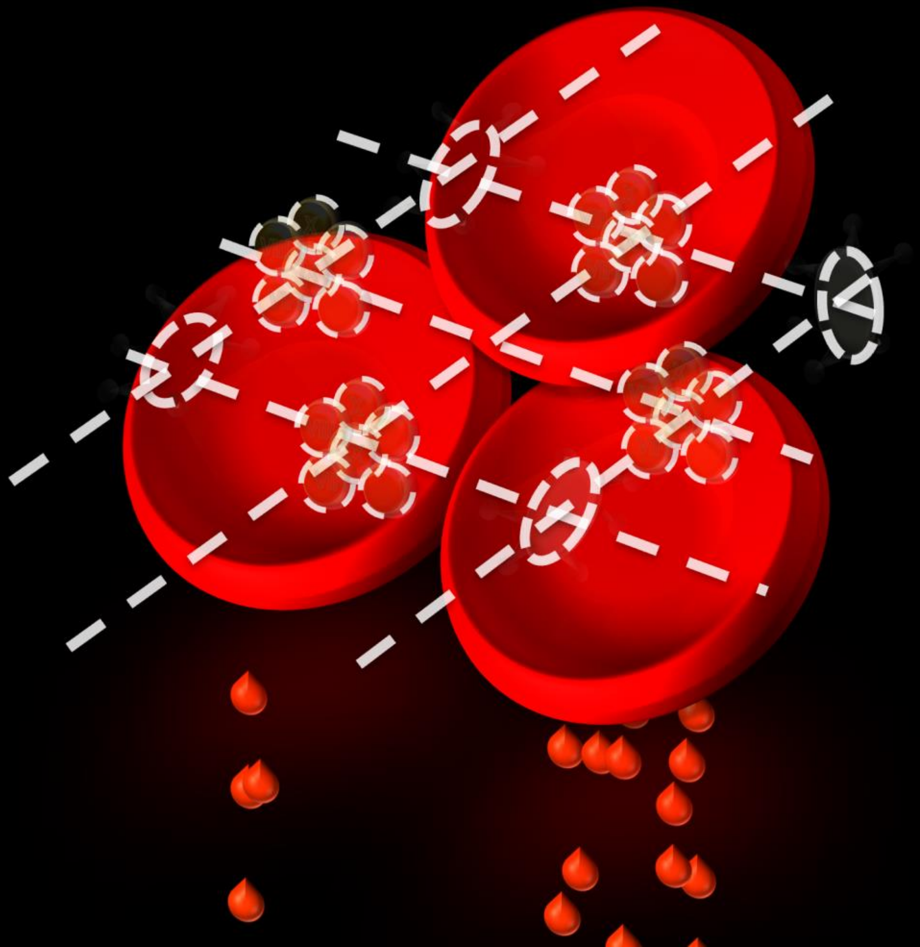

Solution:
Fibrinogen
Platelets
Anti-hyperfibrinolytic
Protamine
Plasmatic factors
No treatment required

Fibrin deficiency

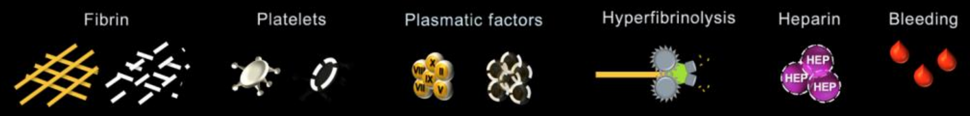

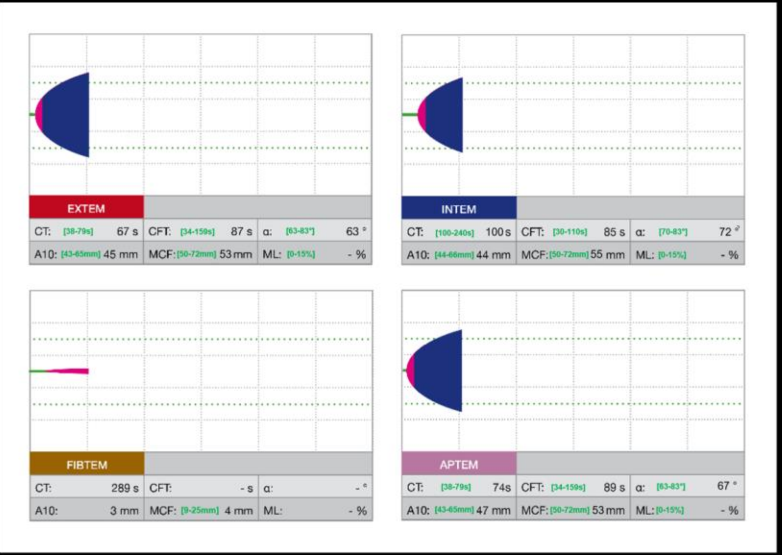

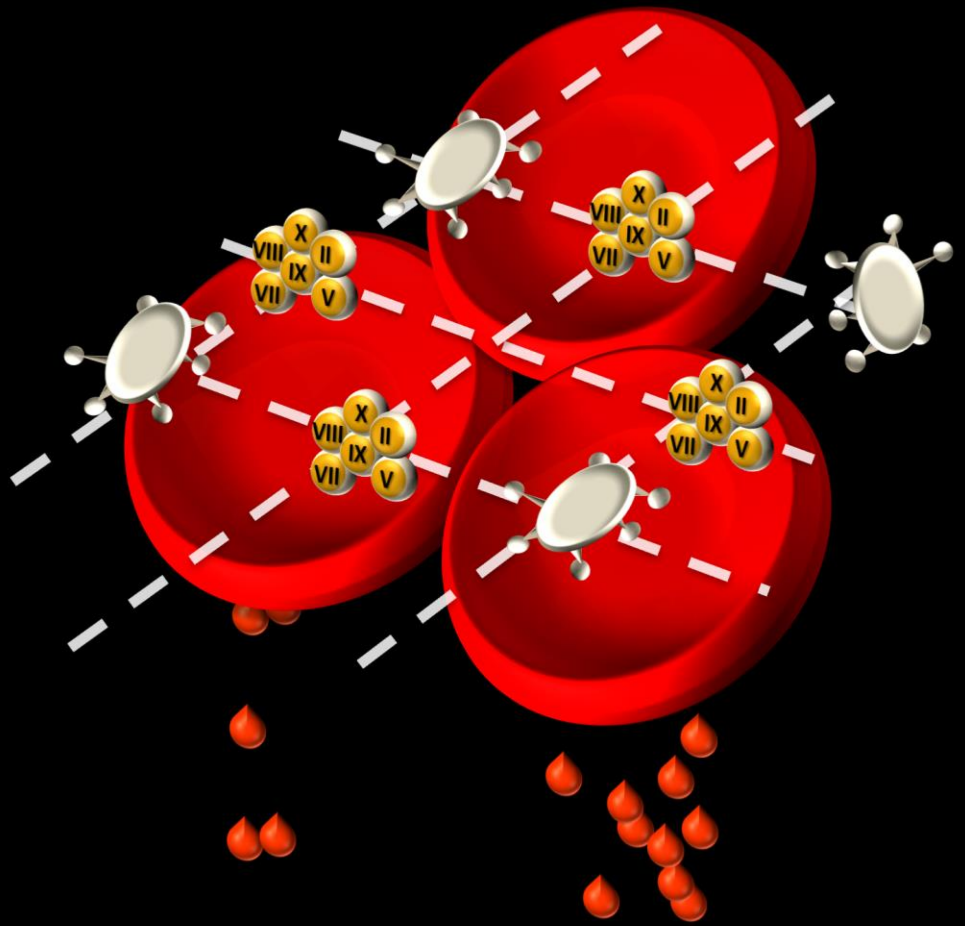

Solution:
Fibrinogen
Platelets
Anti-hyperfibrinolytic
Protamine
Plasmatic factors
No treatment required

Hyperfibrinolysis

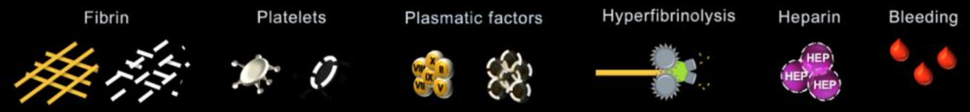

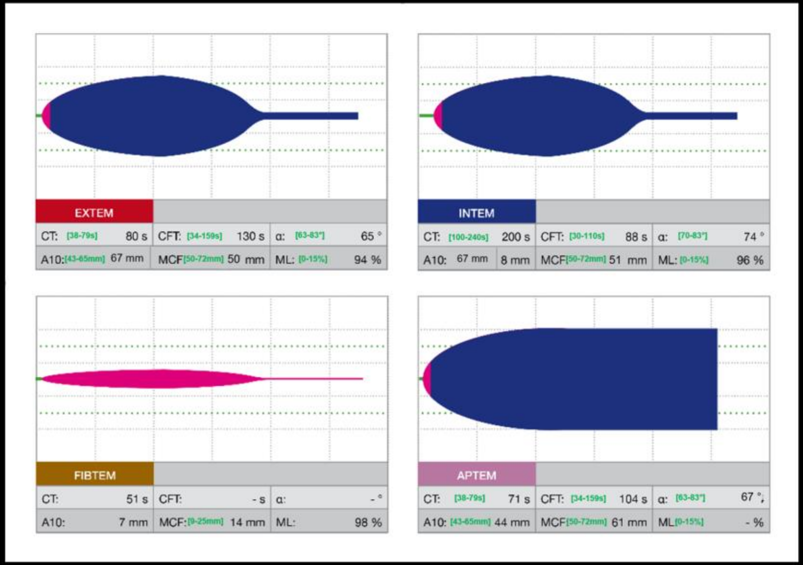

Solution:
Fibrinogen
Platelets
Anti-hyperfibrinolytic
Protamine
Plasmatic factors
No treatment required

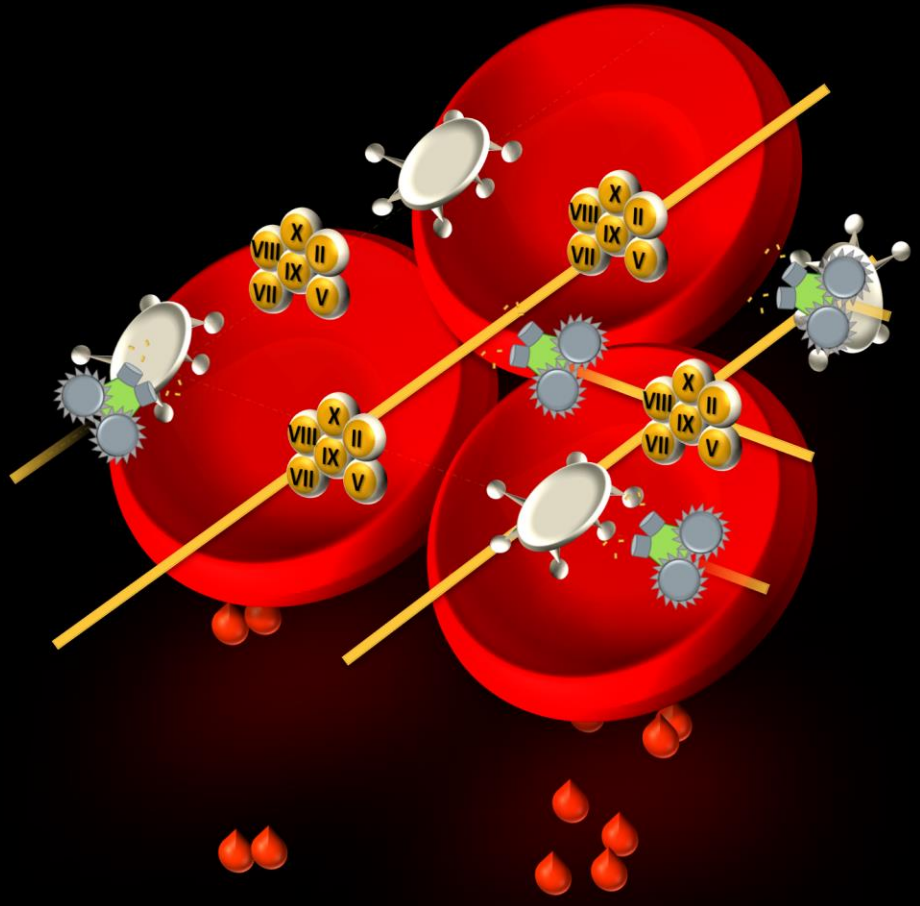

Plasmatic factors deficiency

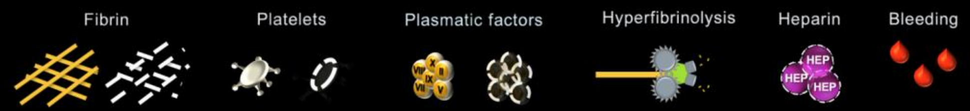

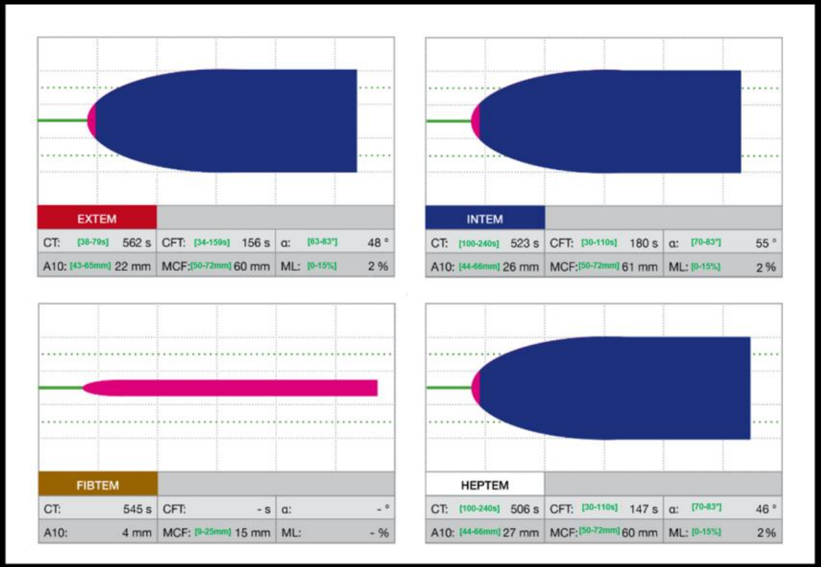

**Solution:**
Fibrinogen
Platelets
Anti-hyperfibrinolytic
Protamine
Plasmatic factors
No treatment required

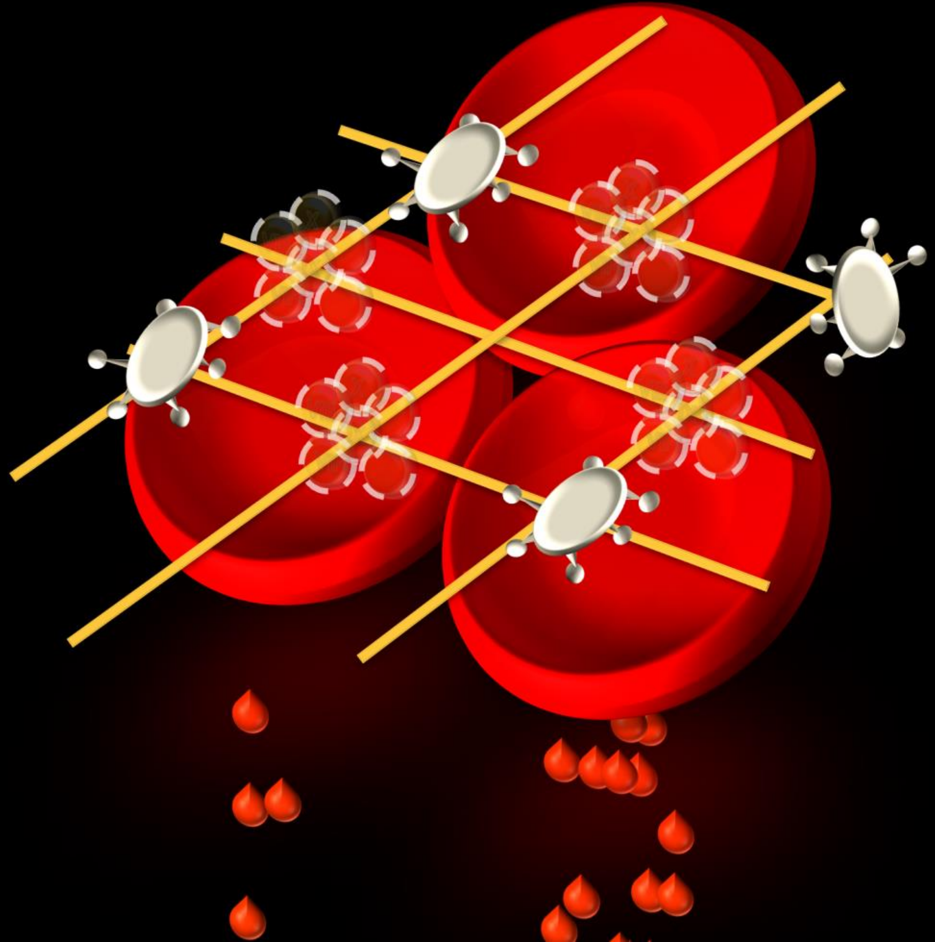

Hypercoagulability

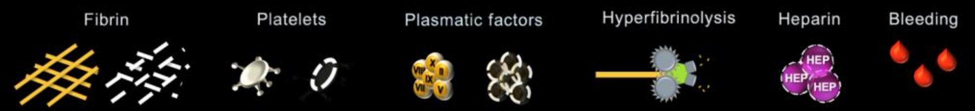

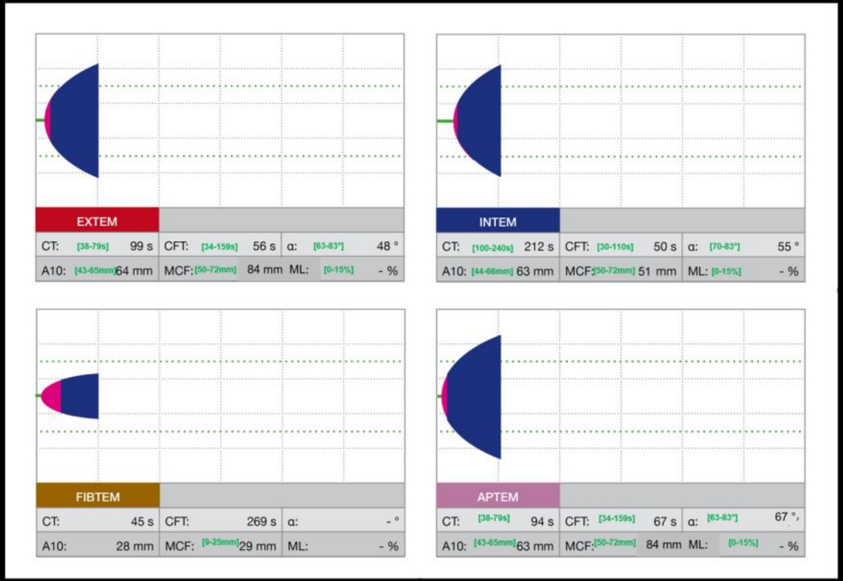

**Solution:**
Fibrinogen
Platelets
Anti-hyperfibrinolytic
Protamine
Plasmatic factors
No treatment required

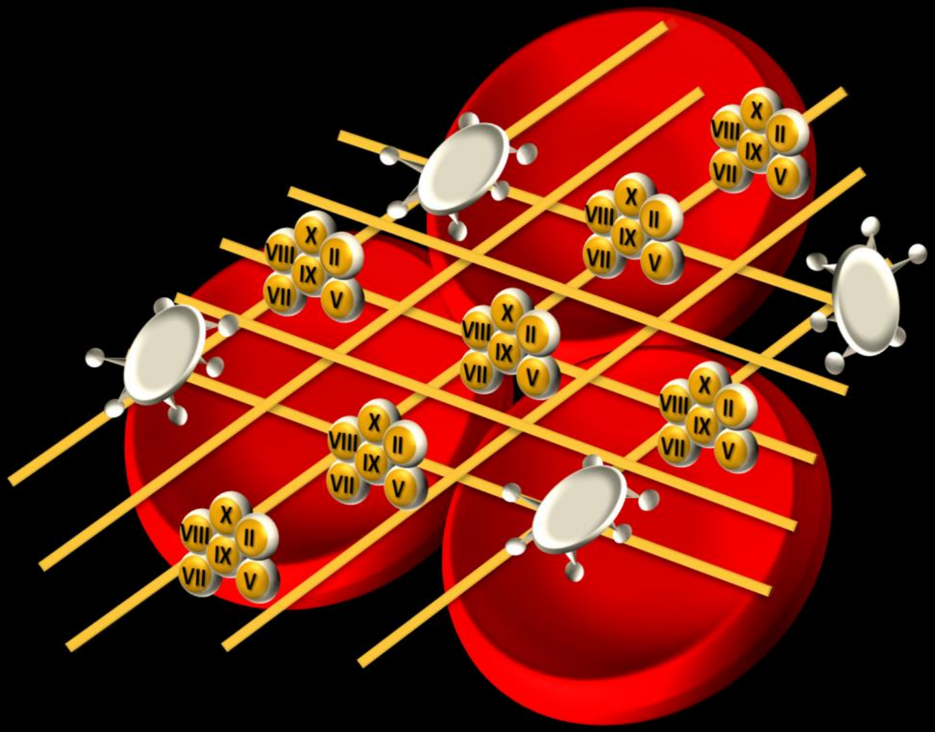

Combined plasmatic factors and fibrin deficiency, and hyperfibrinolysis

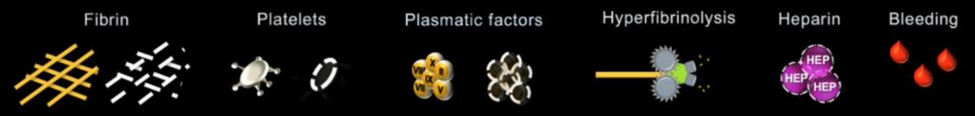

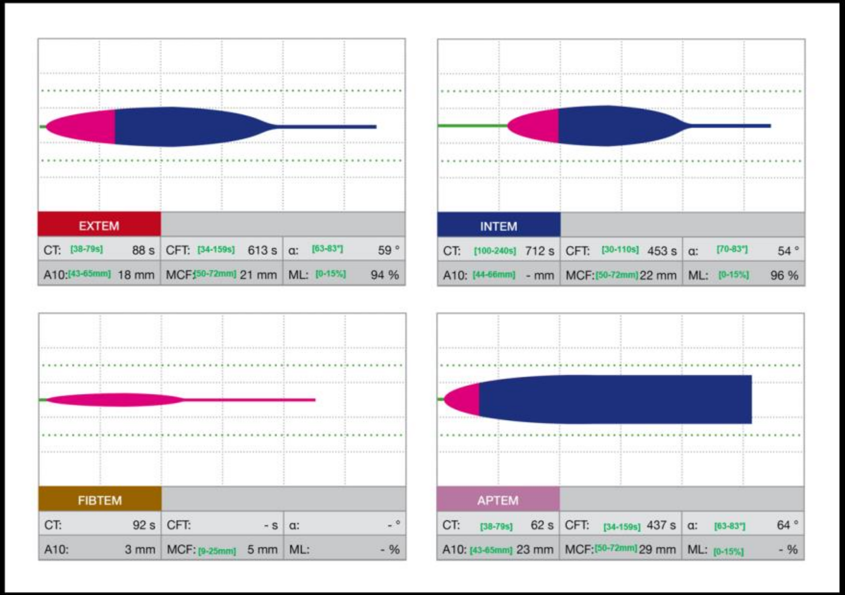

Solution:
Fibrinogen
Platelets
Anti-hyperfibrinolytic
Protamine
Plasmatic factors
No treatment required

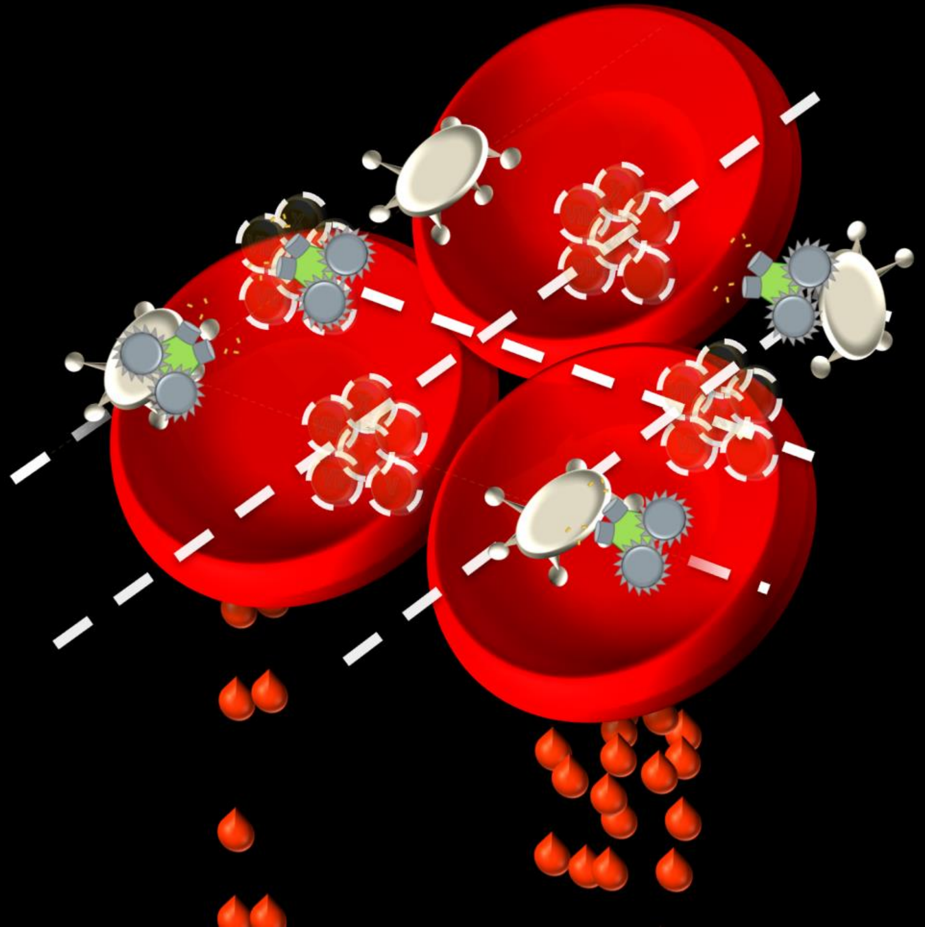

Platelets deficiency

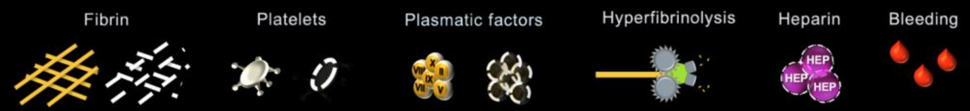

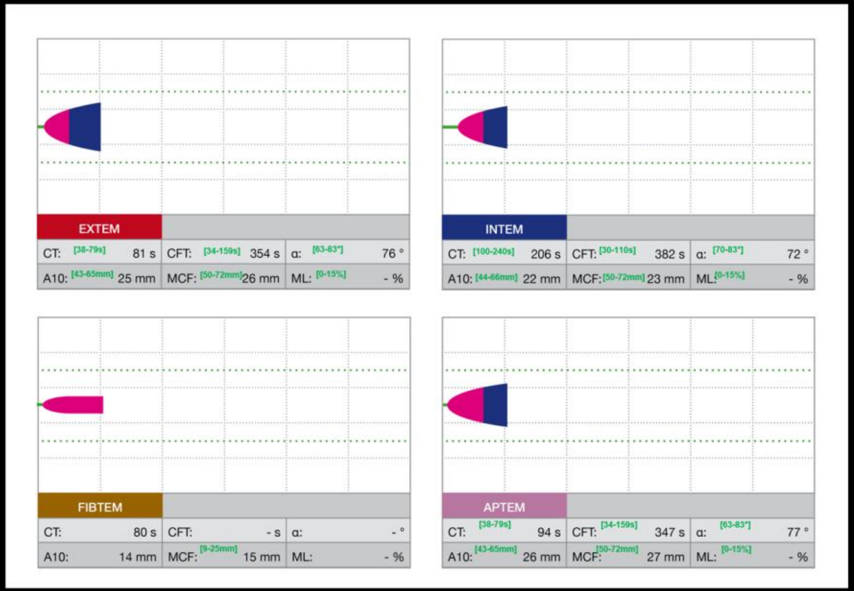

Solution:
Fibrinogen
Platelets
Anti-hyperfibrinolytic
Protamine
Plasmatic factors
No treatment required

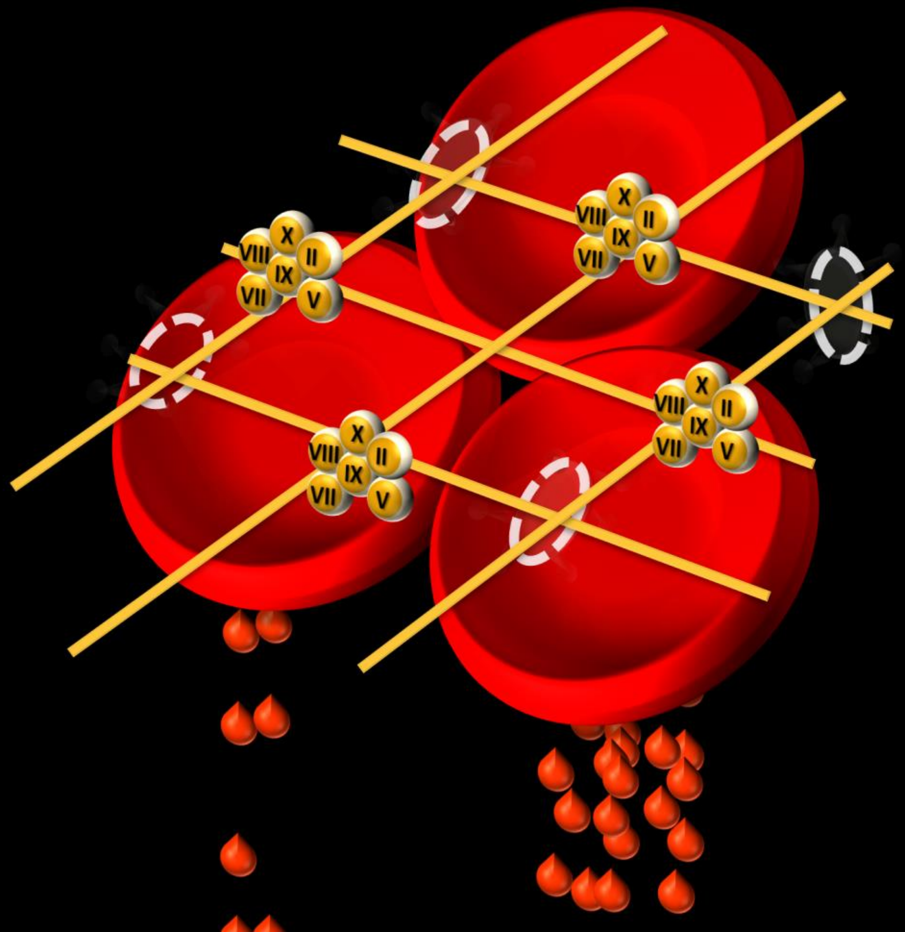

Supplement: Multimedia Appendix 1 [file jmir_v23i5e27124_app1.pdf]
