# Supplementary material for: Effects of an Animated Blood Clot Technology (Visual Clot) on the Decision-Making of Users Inexperienced in Viscoelastic Testing: Multicenter Trial
Source: J Med Internet Res. 2021 May 3;23(5):e27124. doi: 10.2196/27124 (PMC8129883; doi:10.2196/27124)
Supplement: Multimedia Appendix 3 [file jmir_v23i5e27124_app3.pdf]

Effects of an animated blood clot on the decision-making of users inexperienced in viscoelastic testing - Visual Clot technology: a multicenter trial

Sadiq Said[1], Tadzio Raoul Roche[1], Julia Braun[2], Michael Thomas Ganter[3], Patrick Meybohm[4], Johannes Herrmann[4], Kai Zacharowski[5], Eva Rivas[6], Manuel Lopez-Baamonde[6], Florian Jürgen Raimann[5], Florian Piekarski[5], Donat Rudolf Spahn[1], Christoph Beat Nöthiger[1], David Werner Tscholl[1,*]

Appendix 3: Visual Clot algorithm. MCF=Maximum Clot Firmness, ML=Maximum Lysis.

Fibrin deficiency = true	IF "MCF FIBTEM" < 9 mm
Thrombocyte deficiency = true	IF "MCF FIBTEM" 9-25 mm AND "MCF Extem" < 40mm OR "MCF Intem" < 40mm<br>OR<br>IF "MCF FIBTEM" > 25mm AND "MCF Extem" < 50mm OR "MCF Intem" < 50mm<br>OR<br>IF "MCF FIBTEM" < 9mm AND "MCF Extem" < 30mm OR "MCF Intem" < 30mm<br>OR<br>IF "ML INTEM" > 15% OR "ML EXTEM" > 15% OR "ML FIBTEM" > 15% AND "MCF APTEM" < 30mm
Plasmatic factors deficiency = true	IF "CT EXTEM" > 79sec OR "CT INTEM" > 240sec AND "Fibrin deficiency" = false AND "Thrombocyte deficiency" = false AND "Hyperfibrinolysis" = false AND "Hypercoagulability" = false AND "Heparin effect" = false<br>OR<br>IF "CT EXTEM" >100sec OR "CT INTEM" > 260sec.
Hyperfibrinolysis = true	IF "ML EXTEM" > 15% OR "ML INTEM" > 15% OR "ML FIBTEM" > 15% AND "ML APTEM" < 15%
Hypercoagulability = true	IF "MCF EXTEM" > 72mm or "MCF INTEM" > 72mm
Heparin effect = true	IF "CT INTEM" > 240sec AND "CT HEPTEM" < 240sec
