# Supplementary material for: Effects of an Animated Blood Clot Technology (Visual Clot) on the Decision-Making of Users Inexperienced in Viscoelastic Testing: Multicenter Trial
Source: J Med Internet Res. 2021 May 3;23(5):e27124. doi: 10.2196/27124 (PMC8129883; doi:10.2196/27124)
Supplement: Multimedia Appendix 4 [file jmir_v23i5e27124_app4.pdf]

Effects of an animated blood clot on the decision-making of users inexperienced in viscoelastic testing - Visual Clot technology: a multicenter trial.

Version 1.1, Julia Braun, November 16, 2020

Changes in comparison to the former version of this document:

- McNemar test added

1 Introduction

This study compares two possibilities of displaying the results of viscoelastic coagulation tests, ROTEM and Visual Clot, in users without previous experience (medical students and early residents). The report is organised as follows: After a brief explanation of the methods, a description of the data is shown. This is followed by a comparison of the two modalities with respect to performance, the NASA task load index and diagnostic confidence.

2 Methods

For descriptive statistics, we show means, standard deviations as well as medians and interquartile ranges for continuous data and numbers and percentages for categorical data. Our outcome variables are the binary information about performance (correct versus incorrect), the perceived workload (NASA TLX) and the participants' confidence.

For the comparison of ROTEM and Visual Clot, we first apply McNemar's test for an unadjusted comparison of the numbers of correct and incorrect decisions. In a second step, mixed linear and generalized mixed linear models were used to take into account that we have repeated measurements per person and adjust for confounders:

For the binary outcome variables (performance and confidence), we calculated a mixed logistic regression model with random intercept for each participant. For the continuous outcome overall NASA TLX, we calculated a linear mixed model with a random intercept per participant.

Apart from the variable denoting the respective modality (ROTEM versus Visual Clot), all models were adjusted for the following confounders: center, gender and job experience. We did not include the respective scenario because the order in which the scenarios were tackled was completely randomized for each participant and should consequently not have an influence on the overall results.

3 Descriptives

3.1 Participants

Variable	n	Min	q_1	$\tilde{x}$	$\bar{x}$	q_3	Max	s	IQR	#NA
Age	35	24	25.5	28	28.7	31.5	36	3.4	6	0
ROTEM_experience	35	0	0.0	2	3.9	7.0	15	4.4	7	0
ROTEM_theoretical_knowledge	35	0	0.0	0	4.9	10.0	20	7.4	10	0
ROTEM_per_year	35	0	0.0	0	0.2	0.0	6	1.0	0	0

Table 1: Descriptive table per participant - continuous data

Variable	Levels	n	%	$\sum$%
Center	Zurich	7	20.0	20.0
	Winterthur	7	20.0	40.0
	Frankfurt	7	20.0	60.0
	Wurzburg	7	20.0	80.0
	Barcelona	7	20.0	100.0
	all	35	100.0	
Gender	Male	18	51.4	51.4
	Female	17	48.6	100.0
	all	35	100.0	
Job_experience	5th y student	3	8.6	8.6
	6th y student	10	28.6	37.1
	1st y res	19	54.3	91.4
	2nd y res	2	5.7	97.1
	3rd y res	1	2.9	100.0
	all	35	100.0	
Ever_studied_ROTEM	Yes	9	25.7	25.7
	No	26	74.3	100.0
	all	35	100.0	
Ever_studied_VC	Yes	1	2.9	2.9
	No	34	97.1	100.0
	all	35	100.0	
Know_VC	No	35	100.0	100.0
	all	35	100.0	
Q149	neutral	1	2.9	2.9
	agree	9	25.7	28.6
	str. agree	25	71.4	100.0
	all	35	100.0	
Q150	disagree	2	5.7	5.7
	agree	15	42.9	48.6
	str. agree	18	51.4	100.0
	all	35	100.0	
Q151	agree	11	31.4	31.4
	str. agree	24	68.6	100.0
	all	35	100.0	
Q152	neutral	7	20.0	20.0
	agree	11	31.4	51.4

	str. agree	17	48.6	100.0
	all	35	100.0	

Table 2: Descriptive table per participant - categorical data

3.2 Scenarios

Variable	Levels	n	Min	q_1	$\widetilde{x}$	$\bar{x}$	q_3	Max	s	IQR	#NA
Mental_TLX	ROTEM	315	0.0	65.5	80.0	76.1	100.0	100.0	23.4	34.5	0
	VC	315	0.0	12.0	23.0	29.3	43.0	100.0	23.6	31.0	0
	all	630	0.0	22.0	52.5	52.7	82.0	100.0	33.2	60.0	0
Temporal_TLX	ROTEM	315	0.0	18.0	39.0	45.7	75.0	100.0	32.4	57.0	0
	VC	315	0.0	4.5	14.0	18.9	27.5	100.0	18.8	23.0	0
	all	630	0.0	9.0	23.0	32.3	51.8	100.0	29.7	42.8	0
Performance_TLX	ROTEM	315	0.0	68.5	85.0	78.6	97.0	100.0	22.2	28.5	0
	VC	315	0.0	11.0	23.0	26.8	37.0	100.0	21.9	26.0	0
	all	630	0.0	22.0	52.5	52.7	85.0	100.0	34.0	63.0	0
Effort_TLX	ROTEM	315	0.0	50.0	71.0	65.0	85.0	100.0	27.3	35.0	0
	VC	315	0.0	11.0	25.0	29.6	44.0	100.0	23.1	33.0	0
	all	630	0.0	19.0	45.0	47.3	72.8	100.0	30.9	53.8	0
Frustration_TLX	ROTEM	315	0.0	68.5	81.0	78.3	96.0	100.0	19.9	27.5	0
	VC	315	0.0	14.0	25.0	30.9	45.5	100.0	24.8	31.5	0
	all	630	0.0	24.0	58.0	54.6	85.0	100.0	32.7	61.0	0
Overall_TLX	ROTEM	315	13.2	58.6	69.0	68.7	81.1	100.0	17.0	22.5	0
	VC	315	0.0	14.4	25.6	27.1	39.5	79.6	16.3	25.1	0
	all	630	0.0	24.8	47.5	47.9	69.0	100.0	26.7	44.2	0

Table 3: Descriptive table per scenario - continuous data

Variable	Levels	n_{ROTEM}	$\%_{ROTEM}$	$\sum\%_{ROTEM}$	n_{VC}	$\%_{VC}$	$\sum\%_{VC}$	n_{all}	$\%_{all}$	$\sum\%_{all}$
Confidence	Unconfident	277	87.9	87.9	27	8.6	8.6	304	48.2	48.2
	Confident	38	12.1	100.0	288	91.4	100.0	326	51.8	100.0
	all	315	100.0		315	100.0		630	100.0	
Modality	ROTEM	315	100.0	100.0	0	0.0	0.0	315	50.0	50.0
	VC	0	0.0	100.0	315	100.0	100.0	315	50.0	100.0
	all	315	100.0		315	100.0		630	100.0	
Binary_correct	Incorrect	246	78.1	78.1	33	10.5	10.5	279	44.3	44.3
	Correct	69	21.9	100.0	282	89.5	100.0	351	55.7	100.0
	all	315	100.0		315	100.0		630	100.0	

Table 4: Descriptive table per scenario - categorical data

4 Binary performance

	Odds ratio	CI lower	CI upper	p-value
Intercept	0.4	0.14	1.11	0.08
Modality: VC	33.66	21.13	53.64	< 0.0001
Center: Winterthur	1.41	0.62	3.23	0.41
Center: Frankfurt	1.18	0.58	2.43	0.65
Center: Wurzburg	0.82	0.39	1.71	0.60
Center: Barcelona	0.85	0.39	1.87	0.69
Sex. Female	0.63	0.38	1.03	0.06
Exp: 6th y stud	0.76	0.28	2.07	0.59
Exp: 1st y res	0.81	0.33	1.99	0.64
Exp: 2nd y res	0.97	0.3	3.15	0.96
Exp: 3rd y res	1.31	0.26	6.63	0.75

Table 5: Results for mixed logistic regression model for binary performance variable

The percentage of correct answers using ROTEM was 22%, and it was 90% using VC. Applying McNemar's test to those data leads to a p-value of 0.00053 which means that there is very strong evidence for a difference between the two modalities.

In the mixed model, we also see that there is very strong evidence for a difference between the two modalities: The odds of a correct performance are about 33 times as high with the visual clot compared to ROTEM!

5 Confidence

	Odds ratio	CI lower	CI upper	p-value
Intercept	0.03	0	0.26	0.0014
Modality: VC	206.2	93.5	454.75	< 0.0001
Center: Winterthur	4.16	0.77	22.51	0.10
Center: Frankfurt	1.81	0.43	7.66	0.42
Center: Wurzburg	3.29	0.75	14.5	0.12
Center: Barcelona	4.08	0.86	19.39	0.08
Sex. Female	1.02	0.38	2.74	0.96
Exp: 6th y stud	1.42	0.18	11.03	0.73
Exp: 1st y res	1.03	0.16	6.55	0.97
Exp: 2nd y res	0.29	0.03	3.27	0.32
Exp: 3rd y res	9.19	0.36	237.38	0.18

Table 6: Results for mixed logistic regression model for confidence

Also in this case there is a striking difference between the two modalities, with very strong evidence in favour of the visual clot. The odds of being confident are about 200 times higher than with the ROTEM.

6 NASA TLX

	Coefficient	CI lower	CI upper	p-value
Intercept	74.82	63.54	86.1	< 0.0001
Modality: VC	-41.63	-43.91	-39.36	< 0.0001
Center: Winterthur	-10.35	-19.35	-1.35	0.06
Center: Frankfurt	-8.59	-16.39	-0.79	0.07
Center: Wurzburg	0.89	-7.06	8.85	0.85
Center: Barcelona	-3.1	-11.55	5.36	0.54
Sex. Female	1.71	-3.59	7.02	0.59
Exp: 6th y stud	-2.12	-13.15	8.92	0.75
Exp: 1st y res	-3.33	-13.31	6.66	0.58
Exp: 2nd y res	1.46	-11.57	14.49	0.85
Exp: 3rd y res	-12.1	-30.24	6.04	0.27

Table 7: Results for the mixed linear regression model for overall NASA TLX

Also for the overall NASA TLX there is very strong evidence for a difference betwee the two modalities: The NASA TLX was on average about 40 points lower if the VC was used.

R version and packages used to generate this report

R version: R version 3.6.2 (2019-12-12)

Base packages: stats, graphics, grDevices, utils, datasets, methods, base

Other packages: lmerTest, lme4, Matrix, dplyr, stringr, reporttools, xtable, ggplot2, knitr

This document was generated on 2020-11-16 at 16:43.
